# Activation induced deaminase mutational signature overlaps with CpG methylation sites in follicular lymphoma and other cancers

**DOI:** 10.1038/srep38133

**Published:** 2016-12-07

**Authors:** Igor B. Rogozin, Artem G. Lada, Alexander Goncearenco, Michael R. Green, Subhajyoti De, German Nudelman, Anna R. Panchenko, Eugene V. Koonin, Youri I. Pavlov

**Affiliations:** 1 National Center for Biotechnology Information, National Library of Medicine, National Institutes of Health, Bethesda, MD, USA; 2 Novosibirsk State University, Novosibirsk, Russia; 3 Eppley Institute for Research in Cancer and Allied Diseases, University of Nebraska Medical Center, Omaha, NE, USA; 4 Department Microbiology and Molecular Genetics, University of California, Davis, CA, USA; 5 Rutgers Cancer Institute of New Jersey, Rutgers University, New Brunswick, NJ, USA; 6 Department of Neurology and Systems Biology Center, Icahn School of Medicine at Mount Sinai, New York, USA; 7 Departments of Microbiology and Pathology, Biochemistry and Molecular Biology, University of Nebraska Medical Center, Omaha, NE, USA

## Abstract

Follicular lymphoma (FL) is an uncurable cancer characterized by progressive severity of relapses. We analyzed sequence context specificity of mutations in the B cells from a large cohort of FL patients. We revealed substantial excess of mutations within a novel hybrid nucleotide motif: the signature of somatic hypermutation (SHM) enzyme, Activation Induced Deaminase (AID), which overlaps the CpG methylation site. This finding implies that in FL the SHM machinery acts at genomic sites containing methylated cytosine. We identified the prevalence of this hybrid mutational signature in many other types of human cancer, suggesting that AID-mediated, CpG-methylation dependent mutagenesis is a common feature of tumorigenesis.

Analysis of the numerous mutations present in cancer genomes is expected to substantially contribute to our understanding of the causes of malignancy and eventually to the development of personalized treatment plans. DNA sequence contexts of mutations in tumors can provide insights into the mechanisms of mutagenesis in cancer^1^,^2^,^3^. The ‘mutational signature’ approach was introduced in the 1990s^4^,^5^,^6^ and has been successfully applied to delineate the roles of AID and DNA polymerase η in somatic hypermutation in humoral immunity^5^,^7^,^8^, editing APOBEC3s cytosine deaminases in hypermutagenesis in retroviruses^9^ and the formation of dimers versus 6–4 photoproducts in UV- mutagenesis^10^. Recently, this methodology has become popular in the analysis of cancer genomes^3^,^11^. As predicted after the discovery of DNA editing by AID and APOBEC cytosine deaminases^12^, mutations in DNA sequence contexts similar to mutations induced by deaminases in model systems have been found in several types of cancer^2^,^3^,^13^,^14^. Studies of mutations induced by deaminases are facilitated by their unique properties, namely, the ability to produce, *in vitro*, clustered mutations in ssDNA at specific contexts surrounding cytosines^8^,^15^,^16^ and retention of the signatures of deaminase-induced mutagenesis and propensity for clustered mutations, kataegis *in vivo*, in heterologous models where no potential specific cofactors are expected to be present^17^,^18^,^19^,^20^,^21^,^22^,^23^.

Based on DNA sequence context and other approaches, it has been shown that AID, which generates mutations of C-G pairs in the WRC/GYW motif (Fig. 1, upper row, mutated base pair is underlined), could contribute to gastric and haemopoietic cancers^24^,^25^, whereas APOBEC3A (A3A) and A3B (TCW/WGA motif, Fig. 1, fourth row) potentially contribute to breast, lung and many other cancers^17^,^26^,^27^,^28^. A recent report indicates that deaminase-induced clusters of mutations mark signatures of accelerated somatic evolution in cancer gene promoters in lymphoma^29^. Mutational signatures are critical in the analysis and are subject to continuous refinement^30^. Here we describe a novel, unexpected mutational signature of AID deaminase that is linked to DNA CpG methylation. We initially identified the hybrid signature in follicular lymphoma and then in more than a half of all types of human cancers.

## Results and Discussion

We analyzed over 13,000 base substitutions found in follicular lymphoma (FL) in 22 patients (Supplemental Table 1). Mutations at G-C base pairs were 1.5 times more frequent than mutations at A-T pairs; the number of transversions was approximately equal to the number of transitions. The overall pattern of base substitutions in FL has similarities both to the classic distribution of types of changes during spontaneous mutagenesis in humans^31^ and to somatic hypermutation of immunoglobulin genes^7^ (Supplemental Fig. 1). However, the FL mutational spectrum showed alterations in the ratios of transversions in G-C pairs, namely a two-fold relative increase in the fraction of G-C to T-A and a two-fold decrease in the fraction of G-C to C-G transversions, which could be a sign of modulation of processes of DNA damage and translesion DNA synthesis at G-C pairs^32^.

Examination of the DNA sequence context of mutations in FL showed that the bias was caused by a significant excess of substitutions in CpG dinucleotides, with the implication that the mechanism of these mutations is linked to cytosine methylation/demethylation^33^,^34^. Briefly, the analysis was performed as follows. We calculated the excess of mutations in specific motifs using the ratio Fm/Fn, where Fm is the fraction of mutations observed in the particular motif, and Fn is the frequency of the motif in the respective DNA neighborhood (defined as a 120 bp DNA sequence window, Supplemental Dataset S1). A 2.3-fold excess of mutations (defined as described in Materials and Methods) in CG/CG dinucleotides was detected (Table 1, row 1). In contrast, there was no association between mutations and the TCW/WGA motif, indicating that APOBEC1 and APOBEC3 are not involved in mutagenesis in FL (Table 1, row 2). Instead, we detected the signatures of AID and of Pol η (Table 1, rows 3–6), which are known as mutators involved in immunoglobulin genes somatic hypermutation (SHM) at G-C and at A-T base pairs, respectively^35^. Unexpectedly, however, the most strongly over-represented motif was WRCG/CGYW, which is a combination of the AID motif WRC/GYW and the CpG dinucleotide; in contrast, no connection between WRC/GYW and somatic mutations was found in non CpG sites when CpG was masked (Table 1, last three rows). Notably, SHM in immunoglobulin genes shows the opposite trend whereby somatic mutations are substantially underrepresented in CpG-containing motifs^36^. Thus, the mutational process in FL appears to be distinct from the conventional SHM and is likely associated with CpG methylation/demethylation processes. AID deaminates 5-methylcytosine in characteristic AID-target sequence contexts, and the footprint of AID-induced mutagenesis has been found in oncogenes mutated in tumous^37^. Deamination of methylated cytosines by AID and APOBECs^38^ is thought to contribute to a variety of genetic and epigenetic processes^39^,^40^,^41^,^42^, which potentially could be compromised in FL cells, leading to AID-dependent mutagenesis.

The only deviation from this novel mutation pattern in FL was found in 5′UTRs where SHM appears to operate in the “standard immunoglobulin mode” (significant correlation of mutation context with WRCH/DGYW and WA motifs, Supplemental Table 2). Although elevated mutagenesis was observed in CpG dinucleotides and CGYW motif similar to other gene regions, the two processes did not overlap and the hybrid signature was not detected. The 5′UTRs are known to be preferentially targeted by deaminases in active genes^43^,^44^,^45^, therefore the hybrid motif might be masked by numerous AID and other deaminases-induced mutations.

We analyzed AID-related WRC/GYW and WRCG/CGYW motifs for 22 individual FL patient exomes (Supplemental Table 3). A significant excess of both motifs was found for 13 patients. This finding suggests that the mutational processes associated with AID are active in FL to the extent detectable with sensitive statistical tests in samples with limited number of mutations. To determine whether the observed excess of WRCG/CGYW motifs could be a simple consequence of an extremely high mutability of CpG dinucleotides, we compared the relative frequencies of mutations in the WRCG/CGYW motifs and in CpG-containing contexts that do not contain the WRC/GYW motif, namely YCG/CGR and SNCG/CGNS, in different cancer cell lines. In FL and in many other cancers, there was a highly significant excess of mutations in WRCG/CGYW compared to the motifs lacking WRC (Table 2) indicating that the overlap of the AID motif and CpG indeed is the unique mutagenesis signature. In a diverse collection of cancer genomes, we found a significant excess of WRCG/CGYW motifs in two distinct types of blood cancer with the highest representation in the COSMIC data set, as well as in 9 out of 14 analyzed solid tumors from various tissue types, particularly in stomach cancer. Among tissues without an excess of mutated WRCG/CGYW motif, skin has an exceptionally low rate of mutations in this motif, consistent with the previous observations that a different motif (YCG/CGR) is hypermutated in human skin cancers^46^,^47^. Importantly, the signatures characteristic to AID activity are detectable specifically in cancer genomes. For control, we examined the context of somatic mutations in various normal tissues^48^ and did not find any significant excess of AID-related mutable motifs, either CpG-containing or not (Supplemental Tables 4 and 5). The size of these datasets are limited, but power analysis (Materials and Methods) suggested that the absence of any significant excess of AID-related mutable motifs likely reflects genuine biological properties of these samples.

The striking abundance of mutations in WRCG/CGYW motifs in tumors implies that AID is sufficiently active in many human cancer types to skew the mutation distrubition towards the AID WRC/GYW motifs. These observations are in line with the previous findings on the involvement of AID in gastric cancers^25^ and the growing evidence on the role of AID in CpG demethylation in some genomic regions^40^,^49^. We analyzed the mutability of WRC/GYW motifs in various cancer genomes from COSMIC and observed that almost half of the cancer types (6 of the 16) show a significant excess of mutations in these motifs (Table 3). The high mutation prevalence in the “pure” AID motif strongly correlates with that in the hybrid “AID and CpG” motif across the range of cancers. However, the apparent correlation is not perfect and the excess of mutations in WRC/GYW is generally weaker (Fig. 2). The cancers without excess of mutations in WRCG/CGYW (breast, bladder, cervix, lung, skin) show no increased mutability of the WRC/GYW motif either. The difference in the mutability patterns between the two motifs in part can be explained by the greater statistical power of the more informative WRCG/CGYW motifs compared to WRC/GYW motifs. When the involvement of AID is not supported at a statistically significant level through the WRC/GYW motif, it might is still act at CpG dinucleotides causing a significant deviation from the expected mutation frequencies for the WRCG/CGYW motif.

We next compared the expression levels of the AICDA gene, which encodes AID, between the TCGA cohorts. Quartiles and extrema were calculated for each TCGA cohort selected in the study (Supplementary Fig. 2). The observed high variability in AICDA gene expression in B-cell Lymphoma (DLBC) is on par with the observation of widely varyng levels of AICDA expression in peripheral blood mononuclear cells of patients with B-CLL^50^. The expression levels in all other tumor tissues are within the range where definitive conclusions cannot be made based on the data currently available in TCGA (Supplementary Fig. 2). In most tumor cohorts, however, the quantitative profile of the expression values represented by the five numbers summary (and especially the high variability of AICDA expression; see Supplementary Fig. 2) closely follows the one of B-cell lymphoma, which is consistent with the hypothesis presented here.

We next analyzed mutations and the overall level of methylation (% of methylated cytosines or methylation ratio) for 26 patients with malignant lymphoma (https://dcc.icgc.org/projects/MALY-DE, see Methods for details). Consistent with our previous findings (Tables 1 and 3), there is a substantial excess of mutations in WRCG/CGYW and WRC/GYW motifs (4.91 times and 1.53 times, respectively, P < 10^−10^ for both motifs). Analysis of the relative frequencies of mutations in the WRCG/CGYW motifs and in CpG-containing contexts that do not contain WRC/GYW, namely YCG/CGR and SNCG/CGNS, also relealed a highly significant excess (1.5 times, P < 10^−10^) of mutations in motifs containing AID-mutable WRC/GYW, indicating that the overlap of the AID motif and CpG is indeed the signature of mutation process in malignant lymphoma similar to other blood cancers (Table 2). Examination of the association between the methylation ratio and somatic mutations in WRCG/CGYW mutable motifs identified a moderate but significant decrease of methylation in the WRCG/CGYW mutation context. The mean methylation ratios for the WRCG/CGYW mutation positions and non-CGYW mutation positions (YCG/CGR and SNCG/CGNS) were 74.8 and 79.4 respectively (p < 0.0001 according to the sampling test; see Methods for details). The histogram in Fig. 3 shows that the major difference is within the range of methylation ratios of 80 and 100, i.e. in mutation positions with large methylation ratios. This finding is consistent with the hypothesis that AID-dependent demethylation preferentially occurs in WRCG/CGYW mutable motifs so that mutations are one of the outcomes of the multistep demethylation process^37^. No significant difference between the WRCG/CGYW mutable motifs and non- WRCG/CGYW contexts was found for all genomic positions without taking into account somatic mutations in the same set of methylated CpGs (https://dcc.icgc.org/projects/MALY-DE, mean values of the methylation ratio are 73.9 and 74.6, respectively) although the slight overall decrease in the methylation ratio in WRCG/CGYW motifs might have biological implications. These findings are compatible with the hypothesis that AID is involved in demethylation of methylated cytosines during cancer initiation and/or progression.

The analysis of mutations in cancer genomes presented here shows a cancer-specific AID mutational signature that overlaps with the CpG dinucleotide. Thus, AID mutagenesis linked with methylation/demethylation of CpG appears to be a widespread phenomenon in human cancers. The specific mechanisms of the interaction between the CpG (de)methylation and AID–mediated mutagenesis remain to be elucidated. The broader implication of these findings is that epigenetic effects can be directly relevant for somatic mutagenesis in many if not most cancers.

## Methods

**Figure 1:**
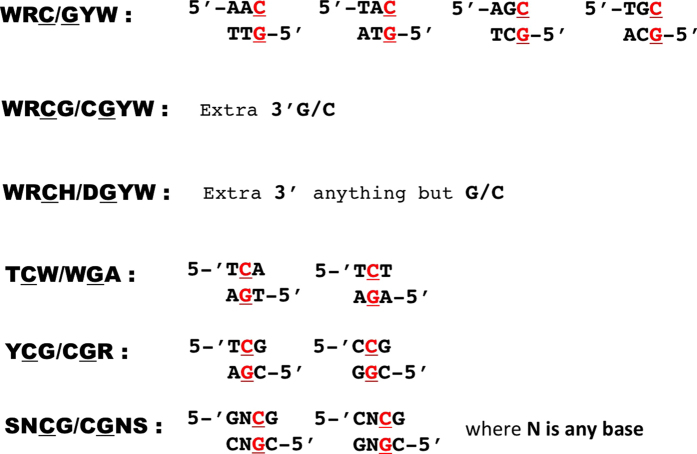
Mutable DNA sequence motifs analyzed in this work. Variants of DNA sequences corresponding to a defined motif (left column, bold) are shown to the right in the double-stranded DNA form. Mutation-prone bases are in red and underlined.

**Figure 2:**
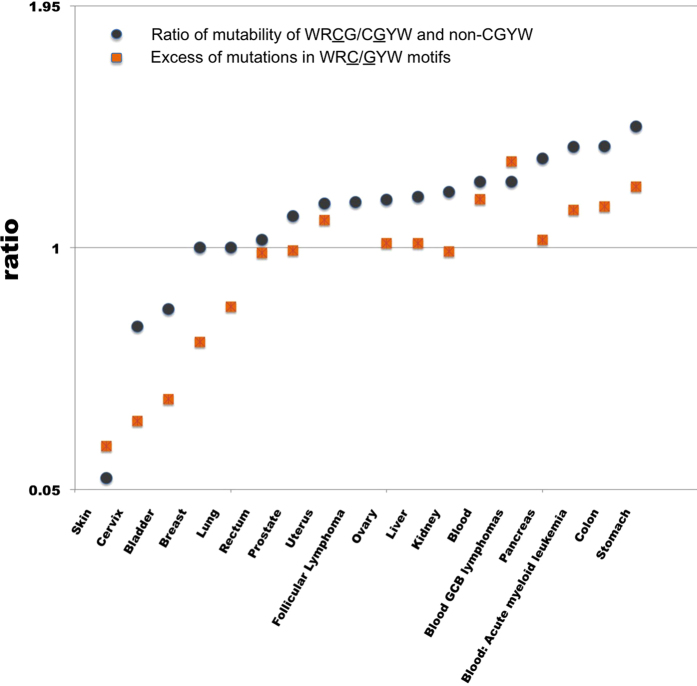
Tumors types with mutation enrichment in the hybrid AID/CpG motif tend to possess an excess of mutations with pure AID signature.

**Figure 3:**
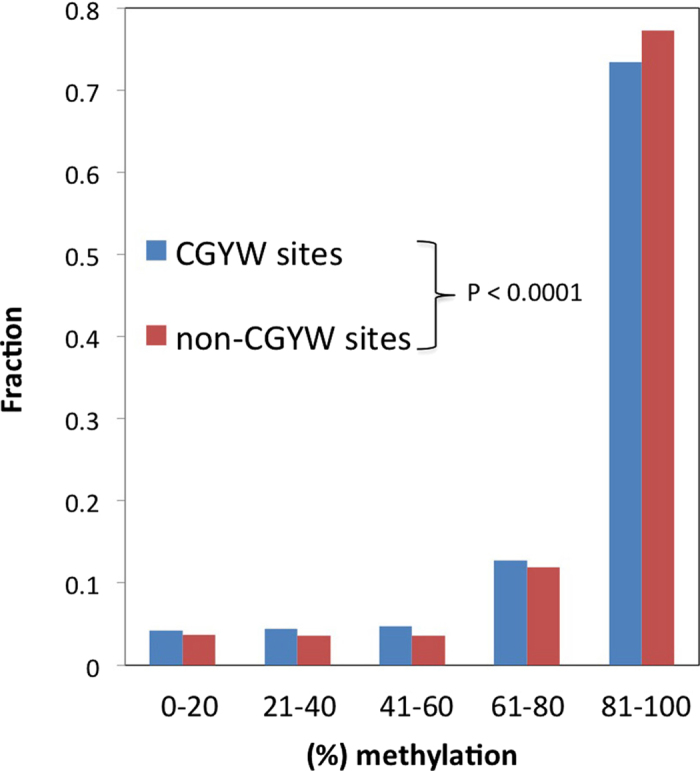
The methylation ratio in WRCG motifs and non- WRCG motifs (YCG/CGR and SNCG/CGNS motifs). The fraction of motifs in each bin (0–20% methylation ratio, 20–40% methylation ratio, etc.) is shown.

The exome sequencing data of 22 follicular lymphoma patients were described previously^51^. DNA sequences surrounding the mutated nucleotide represent the mutation context. We compared the frequency of known mutable motifs for somatic mutations with the frequency of these motifs in the vicinity of the mutated nucleotide. Specifically, for each base substitution the 120 bp sequence centered at the mutation was extracted (the DNA neighborhood). We used only the nucleotides immediately surrounding mutations because AID/APOBEC enzymes are thought to scan a limited area of DNA to deaminate (methyl)cytosines in a preferred motif^26^. This approach does not exclude any given area of the genome in general, but rather uses the areas within each sample where mutagenesis has happened (taking into account the variability in mutation rates across the human genome), and then evaluates whether the mutagenesis in this sample was enriched for AID/APOBEC motifs^26^. This approach was thoroughly tested and a high accuracy of the analysis was shown^26^. The frequency of mutable motifs in the positions of somatic mutations was compared to the frequency of the same motifs in the DNA neighborhood (Fig. 1) using Fisher exact test (2 × 2 table, 2-tail test) and Monte Carlo test (MC, 1-tail test) as previously described^52^,^53^,^54^ (for details see Supplementary Fig. 3). Somatic mutation data from ICGC and TCGA cancer genomic projects were extracted from the Sanger COSMIC Whole Genome Project v75 was downloaded from http://cancer.sanger.ac.uk/wgs. The tissues and cancer types where defined according to primary tumor site and cancer projects. Somatic mutations in various normal tissues were from^48^ (Supplementary Table 5).

We compared magnitude of the difference between the fraction of mutations observed in the mutable motif and the fraction of motifs in surrounding region (effect size) for somatic mutations in normal tissues. For the purpose of this comparison (power analysis), we used a sampling procedure that was repeated 1,000 times. Each sample of somatic mutations from blood and stomach cancers (where significant excess of somatic mutations in WRC/GYW motifs was observed, Tables 2 and 3) had the size equal to those for normal tissues (674 for blood and 49 for stomach, Supplementary Table 5). Analysis of the difference between the fractions showed that the difference for normal mutations was smaller for 98.3% blood cancer samples and for 94.7% stomach cancer samples. Thus the observed effect size (Supplementary Table 5) is likely to reflect biological properties of these samples and is unlikely to be a result of the small sample size at least for somatic mutations from blood and stomach.

For the AICDA gene expression analysis, the normalized version of the RSEM (Broad Institute TCGA Genome Data Analysis Center (2016) Analysis-ready standardized TCGA data from Broad GDAC Firehose 2016_01_28 run. Broad Institute of MIT and Harvard. Dataset. http://doi.org/10.7908/C11G0KM9) was used to analyze the TCGA RNA-Seq datasets from the Broad Genome Data Analysis Center. For each TCGA cohort (Supplementary Fig. 2). The low and upper bounds, median, outliers, and first and third quartiles were retrieved via the FireBrowse RESTful API (http://firebrowse.org/api-docs/) for the tumor and the corresponding normal (when available) tissue samples.

For the analysis of the association between somatic mutations, mutable motifs (WRCG/CGYW) and methylation, datasets for 26 patients with malignant lymphoma (https://dcc.icgc.org/projects/MALY-DE) were used. In the analyzed datasets, the data for all patients were pooled together (the Supplemental Dataset S2 contains the studied set of somatic mutations). Each position is characterized by the methylated/unmethylated read count and the methylation ratio (the number of methylated reads divided by the total number of reads overlapping this position and multiplied by 100). Only positions with more than nine associated reads were included in the analysis. The mean value for mutation positions with (M1) and without WRCG/CGYW (M2) mutable motifs (3620 and 11003 positions, respectively) was calculated. To compare the difference between these two types of positions, methylation ratio values from the larger dataset were randomly sampled until the number of positions was the same as in the smaller dataset. For each sampled dataset, the mean value (M2_sampled) was calculated and the probability P(M1 ≥ M2_sampled) was calculated from 10,000 sampled datasets. The same sampling procedure was used for for all genomic positions without taking into account positions of somatic mutations. Code availability: A set of *ad hoc* programs is available upon request from Igor B. Rogozin (rogozin@ncbi.nlm.nih.gov).

## Additional Information

**How to cite this article**: Rogozin, I. B. *et al*. Activation induced deaminase mutational signature overlaps with CpG methylation sites in follicular lymphoma and other cancers. *Sci. Rep.* **6**, 38133; doi: 10.1038/srep38133 (2016).

**Publisher's note:** Springer Nature remains neutral with regard to jurisdictional claims in published maps and institutional affiliations.

## Supplementary Material

Supplementary Information

Supplementary Dataset 1

Supplementary Dataset 2

## Figures and Tables

**Table 1:** Association between known mutable motifs and the DNA sequence context of somatic mutations in exomes of follicular lymphoma.

Mutable motif	Mutator protein/system	Reference	Excess of mutations in the motif	Type of statistical test*	P- value**
CG/CG	CpG methylation	33	2.3	Fisher	<10^−8^
				Monte-Carlo	<0.001
TCW/WGA	APOBEC1 (A1) or A3A or A3B	27,28,55	0.9	Fisher	NS
				Monte-Carlo	NS
WRC/GYW	AID *in vitro*	8	1.2	Fisher	<10^−7^
				Monte-Carlo	<0.001
WRCH/DGYW	AID component of somatic hypermutation *in vivo* (SHM/AID)	56	1.0	Fisher	NS
				Monte-Carlo	NS
WRCG/CGYW	SHM/AID in FL	This study	2.7	Fisher	<10^−8^
				Monte-Carlo	<0.001
WA/TW	DNA polymerase η (pol η)	7,57	1.1	Fisher	<2 × 10^−5^
				Monte-Carlo	<0.001
All CpG dinucleotides were masked in studied sequences					
TCW/WGA	A1/A3A/A3B		0.8	Fisher	NS
				Monte-Carlo	NS
WRC/GYW	AID *in vitro*		0.9	Fisher	NS
				Monte-Carlo	NS
WA/TW	pol η		1.1	Fisher	<2 × 10^−5^
				Monte-Carlo	<0.001

^*^The correlation was measured using Fisher exact test (Fisher) and Monte Carlo (MC) test. Mutable positions in consensus sequences are underlined (R = A or G; Y = T or C; M = A or C; K = G or T; D = A,T or G; H = A, T, or C; W = A or T, Fig. 1). The excess of mutations in motifs was calculated using the ratio Fm/Fn, where Fm is the fraction of somatic mutations observed in the given mutable motif (the number of mutated motifs divided by the number of mutations), and Fn is the frequency of the motif in the DNA neighborhood of somatic mutations (the number of motif positions divided by the total number of all un-mutated positions in the 120 bp window).

^**^NS, no significant excess.

**Table 2:** Difference between the mutability of AID motifs WRC/GYW with vs without an extra 3′ GC pair (Fig. 1) in various cancer genomes (the Sanger COSMIC Whole Genome Project)*.

Tissue/cancer	Fraction mutated WRCG/CGYW** (total number of motifs)	Fraction mutated YCG/CGR and SNCG/CGNS** (total number of motifs)	P-value, Fisher test
Follicular lymphoma	0.046 (9,438)	0.039 (37,315)	0.005
Blood	0.063 (16,668)	0.050 (71,551)	<10^−10^
Blood: Acute Myeloid Leukemia	0.067 (10,226)	0.048 (43,958)	<10^−10^
Blood: GCB Lymphomas	0.063 (5,033)	0.050 (21,674)	5 × 10^−5^
Breast	0.037 (118,009)	0.037 (449,770)	NS
Bladder	0.022 (55,861)	0.029 (221,217)	NS
Cervix	0.02 (58,896)	0.029 (231,074)	NS
Colon	0.102 (261,993)	0.073 (1,018,315)	<10^−10^
Kidney	0.039 (45,835)	0.032 (179,222)	<10^−10^
Liver	0.036 (115,921)	0.030 (480,198)	<10^−10^
Lung	0.036 (221,301)	0.036 (872,132)	NS
Ovary	0.044 (33,436)	0.037 (132,132)	<10^−10^
Pancreas	0.077 (60,409)	0.057 (242,824)	<10^−10^
Prostate	0.072 (27,084)	0.064 (106,577)	3 × 10^−5^
Rectum	0.086 (46,433)	0.068 (177,479)	<10^−10^
Skin	0.004 (201,866)	0.042 (824,249)	NS
Stomach	0.090 (204,610)	0.061 (802,363)	<10^−10^
Uterus	0.054 (84,699)	0.046 (317,894)	<10^−10^

Tissue types without significant correlation (taking into account the Bonferroni correction for multiple tests) between the motif and somatic mutations are underlined. “Fraction mutated CGYW” and “Fraction mutated YCG/CGR and SNCG/CGNS” are fractions of mutated motifs (the number of the mutated motifs divided by the total number of motifs in the analyzed data set). Absence of significant excess of mutations in CGYW/WRCG (NS, no significant excess) indicates that there is no connection between mutagenesis of CG and GYW motifs.

^**^Total number of motifs are in brackets.

**Table 3:** Preferential mutability of WRC/GYW and somatic mutations in various cancer Whole Genomes and Whole Exomes (the Sanger COSMIC Whole Genome Project)*.

Tissue	Fraction of mutations observed in the mutable motif	Fraction of motifs in surrounding regions	Excess of mutations in the motif	P-value Fisher exact test
Blood	0.265 (10,633)	0.222 (630,338)	1.19	<10^−10^
Blood: Acute Myeloid Leukemia	0.254 (6,844)	0.221 (405,754)	1.15	<10^−10^
Blood: GCB Lymphomas	0.296 (2,747)	0.221 (165,805)	1.34	<10^−10^
Breast	0.146 (85,203)	0.230 (4,877,914)	0.64	NS
Bladder	0.093 (38,750)	0.229 (2,247,271)	0.41	NS
Cervix	0.074 (41,454)	0.229 (2,399,601)	0.32	NS
Colon	0.257 (175,109)	0.221 (8,986,392)	1.16	<10^−10^
Kidney	0.225 (32,382)	0.228 (1,875,298)	0.99	NS
Liver	0.226 (74,161)	0.222 (4,426,455)	1.02	NS
Lung	0.174 (180,284)	0.226 (9,867,123)	0.77	NS
Ovary	0.229 (22,340)	0.225 (1,309,849)	1.02	NS
Pancreas	0.227 (35,165)	0.220 (2,112,760)	1.03	2 × 10^−3^
Prostate	0.22 (13,036)	0.222 (775,226)	0.99	NS
Rectum	0.223 (23,330)	0.226 (1,343,391)	0.99	NS
Skin	0.050 (198,098)	0.224 (8,986,960)	0.22	NS
Stomach	0.274 (115,652)	0.221 (6,977,875)	1.24	<10^−10^
Uterus	0.254 (55,999)	0.229 (3,212,849)	1.11	<10^−10^

Tissue types without significant correlation (taking into account the Bonferroni correction for multiple tests) between the motif and somatic mutations are underlined. The excess of mutations in motifs was calculated using the ratio Fsm/Fc, where Fsm is the fraction of somatic mutations observed in the studied mutable motif (the number of mutated motifs divided by the number of mutations), and Fc is the frequency of the motif in the DNA context of somatic mutations (the number of motif positions divided by the total number of all un-mutated positions in surrounding regions).

^*^Absence of significant excess of mutations in WRC/GYW (NS, no significant excess) suggests that there is no connection between mutagenesis and WRC/GYW motifs.
