# Supplementary material for: Activation induced deaminase mutational signature overlaps with CpG methylation sites in follicular lymphoma and other cancers
Source: Sci Rep. 2016 Dec 7;6:38133. doi: 10.1038/srep38133 (PMC5141443; doi:10.1038/srep38133)
Supplement: Supplementary Information [file srep38133-s1.pdf]

Supplemental information files for:

Activation induced deaminase mutation signature overlaps with CpG methylation in follicular lymphomas and other cancers

Igor B. Rogozin , Artem G. Lada, Alexander Goncearenco, Michael R. Green, Subhajyoti De, German Nudelman, Anna R. Panchenko, Eugene V. Koonin and Youri I. Pavlov

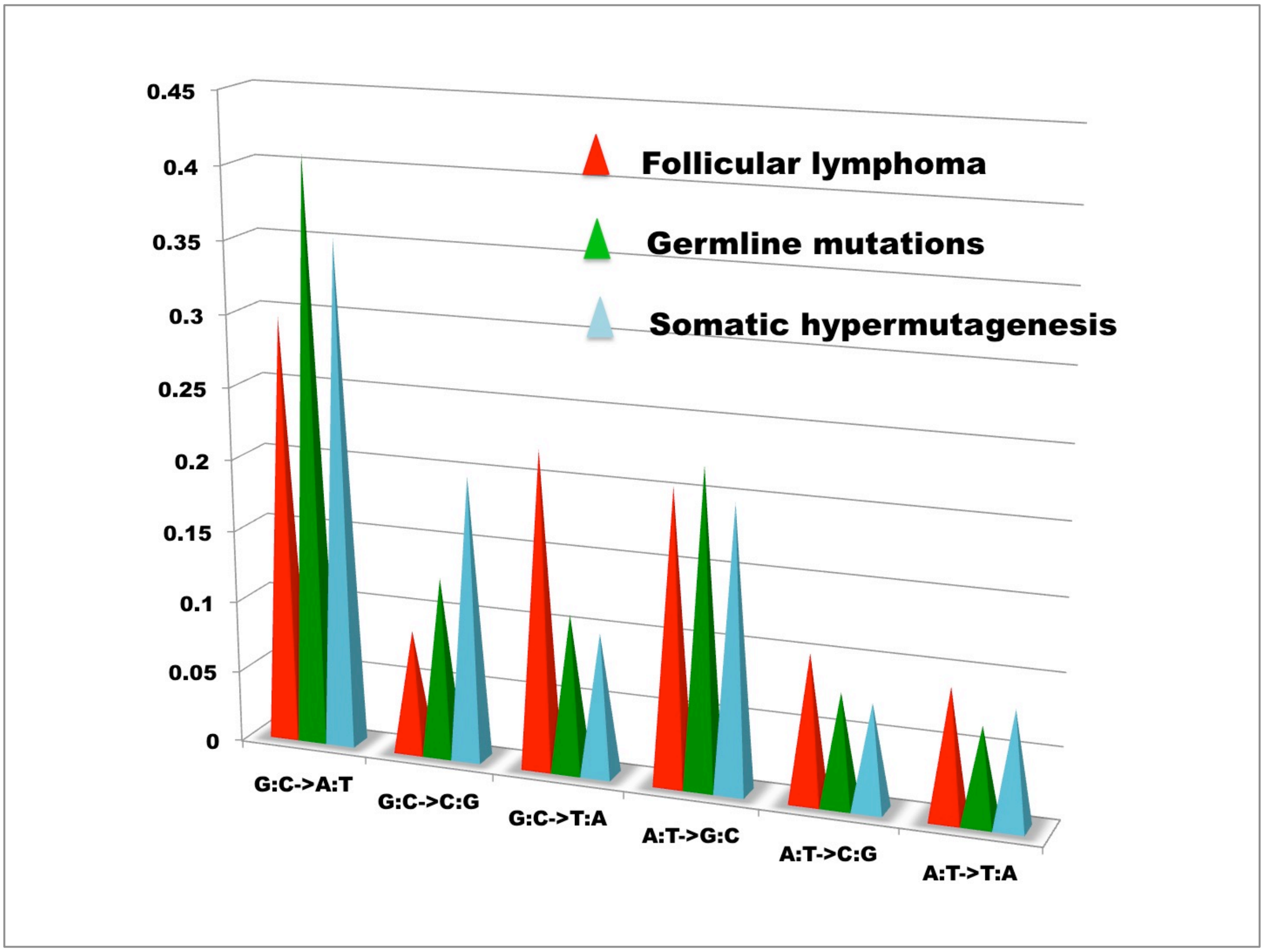

Supplemental Figure 1. Mutation types in FL with human spontaneous mutations and immunoglobulin somatic mutations.

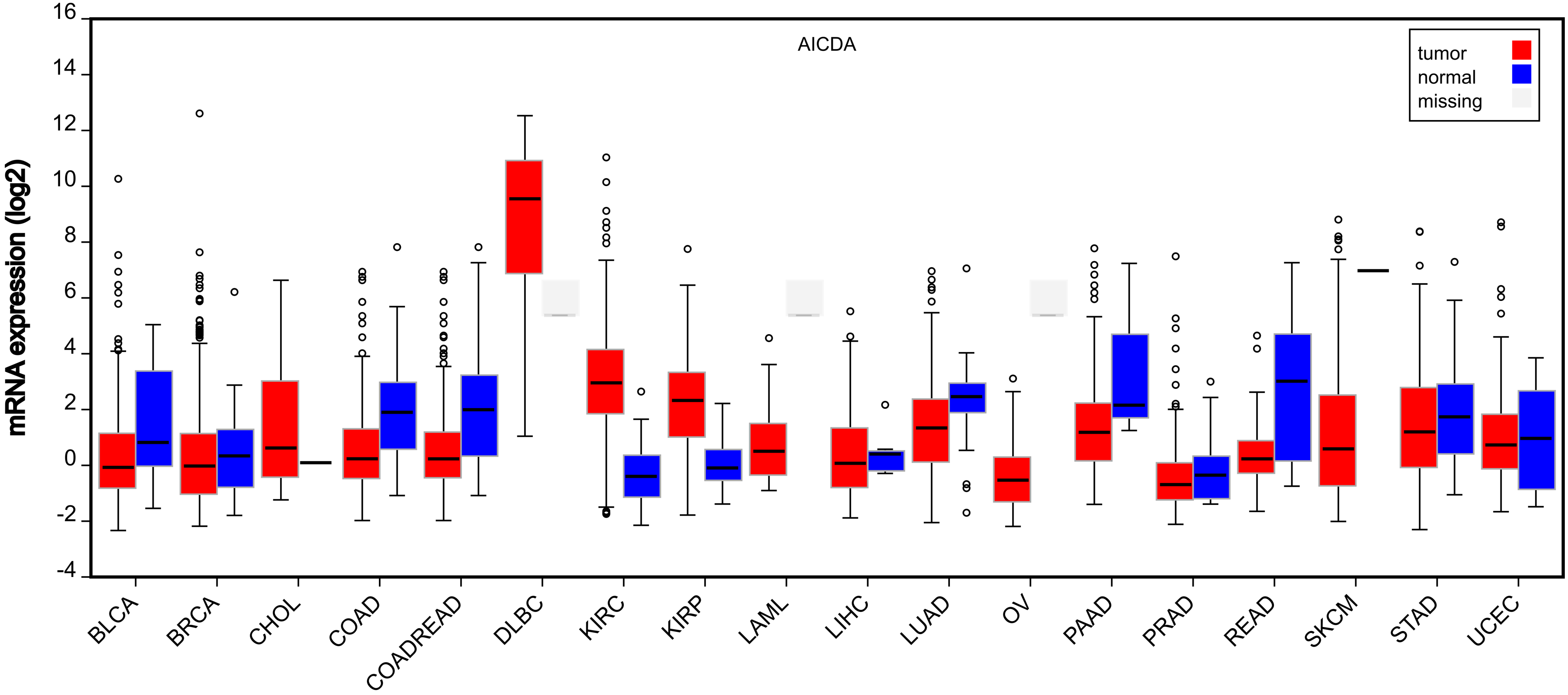

Supplementary Figure 2. Summary of RNA-Seq data for TCGA cohorts selected in the study. The boxplot summary includes the following statistics: the minimum value, the 25th percentile (known as Q1), the median, the 75th percentile (Q3), and the maximum value. The following tissues were analyzed: Bladder urothelial carcinoma (BLCA), Breast invasive carcinoma (BRCA), Cervical and endocervical cancers (CESC), Cholangiocarcinoma (CHOL), Colon adenocarcinoma (COAD), Colorectal adenocarcinoma (COADREAD), Lymphoid Neoplasm Diffuse Large B-cell Lymphoma (DLBC), Kidney renal clear cell carcinoma

(KIRC), Kidney renal papillary cell carcinoma (KIRP), Acute Myeloid Leukemia (LAML), Liver hepatocellular carcinoma (LIHC), Lung adenocarcinoma (LUAD), Ovarian serous cystadenocarcinoma (OV), Pancreatic adenocarcinoma (PAAD), Prostate adenocarcinoma (PRAD), Rectum adenocarcinoma (READ), Skin Cutaneous Melanoma (SKCM), Stomach adenocarcinoma (STAD), Uterine Corpus Endometrial Carcinoma (UCEC).

(A)

----atCtG**CG**aa**C** **G** Ctt**CG**tGtta-----

----ttt**CG**aCCtt C CttCCCtaaa-----

----**CGCG**ttatta **C G**taaatttCC-----

Context ^ Context

^

Position of mutation

C:G shown in capital letters, CpG shown in bold, hotspot positions are underlined

Fsm = 2/3 (2 mutations in CG, 1 in non-CG)

Fc = 12/26 (12 C:G nucleotides belongs to CpG, 26 C:G positions in total)

Fisher 2x2 table

**2**	**1**
**12**	**14**

(B)

```
----atCtGCGaaC G CttCGtGtta-----

----tttCGaCCtt C CttCCCtaaa-----

----CGCGttatta C GtaaatttCC-----

     Context   ^   Context

               ^

               Position of mutation
```

C:G shown in capital letters, CpG shown in bold, hotspot positions are underlined, CGYW/WRCG marked in yellow

Fraction mutated CGYW = 2/4 = 0.5 (total number of motifs = 4)

Fraction mutated CGR/CGNS (non-CGYW) = 1/6 = 0.17 (total number of motifs = 6)

Fisher 2 x 2 table

2	2
1	5

Supplementary Figure 3. Statistical analysis of mutable motifs in sites of somatic mutations and surrounding regions.

(A) The excess of mutations in motifs was calculated using the ratio Fm/Fn, where Fm is the fraction of somatic mutations observed in the given mutable motif (the number of mutated motifs divided by the number of mutations), and Fn is the

frequency of the motif in the DNA neighborhood of somatic mutations (the number of motif positions divided by the total number of all un-mutated positions in the 120 bp window). (B) “Fraction mutated CGYW” and “Fraction mutated CGR and CGNS” are fractions of mutated motifs (the number of the mutated motifs divided by the total number of motifs in the analyzed data set).

Supplemental Table 1. Types of base-substitution somatic mutations in follicular lmphoma exomes [1].

Types of base substitutions	Number
Transitions:	
G:C->A:T	3915
A:T->G:C	2711
Total transitions	6626
Transversions:	
G:C->C:G	1133
G:C->T:A	2929
A:T->C:G	1353
A:T->T:A	1199
Total transversions	6614
Mutations at G:C pairs	7977
Mutations in A:T pairs	5263
Total base substitutions	13240

Supplemental Table 2. Variations of the association between mutable motifs and DNA sequence context of mutations in different gene regions.

Mutable motif (n)	Mutator protein/system	Excess of mutations in the motif	Type of statistical test*	P value
5'UTRs (total number of mutations in C+G = 248, in A+T = 144)				
CG/GC (74)	CpG methylation	1.6	Fisher	$<10^{-5}$
			Monte-Carlo	<0.001
TCW/WGA (28)	A1/A3A/A3B	0.8	Fisher	NS
			Monte-Carlo	NS
WRCH/DGYW (67)	SHM/AID	1.5	Fisher	$<10^{-4}$
			Monte-Carlo	<0.001
WRCG/CGYW (11)	SHM/AID	1.6	Fisher	NS
			Monte-Carlo	NS
WA/TW (70)	pol η	1.2	Fisher	0.011
			Monte-Carlo	0.013
Gene bodies (total number of mutations in C+G = 5376, number of mutations in A+T = 3332)				
CG/GC (1190)	CpG methylation	2.4	Fisher	$<10^{-8}$
			Monte-Carlo	<0.001
TCW/WGA (718)	A1/A3A/A3B	0.9	Fisher	NS
			Monte-Carlo	NS
WRCH/DGYW (934)	SHM/AID	0.8	Fisher	NS

			Monte-Carlo	NS
WRCG/CGYW (233)	SHM/AID	2.5	Fisher	$<10^{-8}$
			Monte-Carlo	<0.001
WA/TW (1836)	pol η	1.1	Fisher	$<10^{-4}$
			Monte-Carlo	<0.001
3'UTRs (total number of mutations in C+G = 602, in A+T = 463)				
CG/GC (83)	CpG methylation	2.1	Fisher	$<10^{-5}$
			Monte-Carlo	<0.001
TCW/WGA (97)	A1/A3A/A3B	0.9	Fisher	NS
			Monte-Carlo	NS
WRCH/DGYW (130)	SHM/AID	0.9	Fisher	NS
			Monte-Carlo	NS
WRCG/CGYW (16)	SHM/AID	1.3	Fisher	0.023
			Monte-Carlo	0.024
WA/TW (267)	pol η	1.	Fisher	NS
			Monte-Carlo	NS

Supplemental Table 3. Analysis of somatic mutations in AID-related motifs in 22 FL patients.

			WRC/GYW			WRCG/CGYW vs. WRCH/DGYW		
Patient	#mutations*		#mutations in GYW	Excess	P-value	#mutations in CGYW	Excess	P-value
PJ040	311		40	.79	NS	7	.72	NS
PJ041	466		67	1.08	.25	15	1.08	.42
PJ128	473		60	.81	NS	17	.91	NS
PM001	614	+	121	1.55	.00000018	24	1.27	.13
PM005	252	+	46	1.32	.02	10	1.12	.41
PM011	1843	+	225	1.29	.000016	80	1.28	.012
PM012	359	+	50	1.29	.027	13	1.25	.23
PM013	322		40	.83	NS	9	.99	NS
PM016	296	+	56	1.55	.0003	15	1.66	.028
PM017	219	+	37	1.40	.017	7	.82	NS
PM018	758	+	136	1.29	.0009	36	1.55	.004
PM019	436		67	1.19	.07	12	1.05	.48
PM020	659	+	100	1.41	.0001	27	1.30	.09
PM021	480	+	81	1.39	.0009	6	.57	NS
PM022	287	+	49	1.24	.05	10	.92	NS
PM023	218		34	1.16	.20	5	.76	NS
PM024	572	+	148	1.80	<10-8	28	1.68	.003
PM025	236	+	52	1.57	.00036	18	1.92	.005
PM026	1547		159	.49	NS	22	.52	NS
PM027	1117		122	.82	NS	23	1.00	NS
PM028	1075		122	1.02	.41	21	1.06	.41
PM029	700	+	109	1.27	.004	22	1.31	.11

* - "+" means that at least one P value is less or equal 0.05.

Supplemental Table 4. No differences are detected between spontaneous mutability of CGYW and non-CGYW (**CGR and CGNS)** in normal tissues* [2].

Tissue	Fracton of mutated CGYW** (total number of motifs)	Fraction of mutated CGR and CGNS** (total number of motifs)	P Fisher
Blood	0.025 (961)	0.035 (4338)	NS
Bladder	0.027 (75)	0.032 (383)	NS
Lung	0.035 (387)	0.034 (1807)	NS
Prostate	0.015 (134)	0.024 (682)	NS
Stomach	0.013 (78)	0.037 (306)	NS

* No significant correlation (taking into account the Bonferroni correction for multiple tests) between the motif and somatic mutations was detected.

** “Fraction mutated CGYW” and “Fraction mutated CGR and CGNS” are fractions of mutated motifs (the number of the mutated motif divided by the total number of motifs in the studied dataset). Absence of significant excess of mutations in CGYW/WRCG (NS, no significant excess) suggests that there is no connection between mutagenesis of CG and GYW motifs. Total number of motifs are in brackets

Supplemental Table 5. No association is detected between the AID mutable motif WRC/GYW and somatic mutations in the most normal tissues [2].

Tissue	Fraction of mutations observed in the mutable motif	Fraction of motifs in surrounding regions	Excess of mutations in the motif	P fisher
Blood	0.168 (674)	0.276 (41,738)	0.61	NS
Bladder	0.205 (539)	0.202 (2,672)	1.01	NS
Lung	0.260 (247)	0.269 (15,802)	0.96	NS
Prostate	0.182 (65)	0.233 (4,370)	0.78	NS
Stomach	0.065 (49)	0.256 (3,166)	0.24	NS

Tissue types without significant correlation (taking into account the Bonferroni correction for multiple tests) between the motif and somatic mutations are underlined. The excess of mutations in motifs was calculated using the ratio Fsm/Fc, where Fsm is the fraction of somatic mutations observed in the studied mutable motif (the number of mutated motifs divided by the number of mutations), and Fc is the frequency of the motif in the DNA context of somatic mutations (the number of motif positions divided by the total number of all un-mutated positions in surrounding regions). The absence of significant excess of mutations in WRC/GYW suggests that there is no connection between mutagenesis and GYW/WRC motifs.

References.

1 Green, M. R. *et al.* Mutations in early follicular lymphoma progenitors are associated with suppressed antigen presentation. *Proceedings of the National Academy of Sciences of the United States of America* **112**, E1116-1125, doi:10.1073/pnas.1501199112 (2015).

2 Yadav, V. K., DeGregori, J. & De, S. The landscape of somatic mutations in protein coding genes in apparently benign human tissues carries signatures of relaxed purifying selection. *Nucleic acids research* **44**, 2075-2084, doi:10.1093/nar/gkw086 (2016).
